# Citizen scientists reliably count endangered Galápagos marine iguanas from drone images

**DOI:** 10.1038/s41598-025-08381-9

**Published:** 2025-07-24

**Authors:** Andrea Varela-Jaramillo, Christian Winkelmann, Andrés Mármol-Guijarro, Juan M. Guayasamin, Gonzalo Rivas-Torres, Sebastian Steinfartz, Amy MacLeod

**Affiliations:** 1 Institute of Biology, Molecular Evolution and Systematics of Animals, University of Leipzig, Leipzig, Saxony, Germany; 2 3Diversity, Quito, Pichincha, Ecuador; 3 Eberswalde University for Sustainable Development, Eberswalde, Brandenburg, Germany; 4 Laboratorio de Biología Evolutiva, Colegio de Ciencias Biológicas y Ambientales COCIBA, Instituto Biósfera, Universidad San Francisco de Quito USFQ, Quito, Pichincha Ecuador; 5 Galápagos Science Center, GSC, San Cristóbal, Galápagos, Ecuador; 6 Wildlife Ecology and Conservation, University of Florida, Gainesville, FL United States of America

**Keywords:** Aerial imagery, Citizen science, UAVs, Wildlife monitoring, Zooniverse, Ecology, Zoology

## Abstract

Population surveys are essential for conservation, but are often resource-intensive. Modern technologies, like drones, facilitate data collection but increase the analysis burden. Citizen Science (CS) offers a solution by engaging non-specialists in data analysis. We evaluated CS for monitoring marine iguanas, focusing on volunteers’ accuracy in detecting and counting individuals in aerial images. During three phases of our Zooniverse project, over 13,000 volunteers contributed 1,375,201 classifications from 57,838 images; each classified up to 30 times. Using a Gold Standard dataset of expert counts from 4,345 images, we evaluated optimal aggregation methods for CS-inputs. Volunteers achieved 68–94% accuracy in detection, with more false negatives than false positives. The standard ‘majority vote’ aggregation approach (where the answer given by the majority of individual inputs is selected) produced less accuracy than when a minimum threshold of five volunteers (from the total independent classifications) was used. Image quality significantly influenced accuracy; by excluding suboptimal pilot-phase data, volunteer counts were 91–92% accurate. HDBSCAN clustering yielded the best results. We conclude that volunteers can accurately identify and count marine iguanas from drone images, though there is a tendency for undercounting. However, even CS-based data analysis remains relatively resource-intensive, underscoring the need to develop an automated approach.

**Supplementary Information:**

The online version contains supplementary material available at 10.1038/s41598-025-08381-9.

## Introduction

Population monitoring is a central task in conservation work, since the information gathered forms the foundation of wildlife management plans. However, for many species, collecting such data by traditional means often requires more resources and time than are available. Of the 81% of vertebrate species assessed by the IUCN, around 14% are currently data deficient (DD)^1^ with unknown population status and distribution range being the key reason for that deficiency in many groups^2^. Modern approaches employing new technologies — such as drones for aerial surveys — significantly reduce monitoring effort and surveyor risk while simultaneously allowing access to remote regions^3–5^. However, along with the rise in popularity of image-based approaches, comes an increased burden for data analysis, which presents a significant challenge for conservation practitioners.

One increasingly popular approach that can ease this bottleneck and thus ensure the long-term future of image-based approaches, involves mobilizing non-specialist help in the form of Citizen Scientists (CS; also referred to as volunteers). Whilst there are already numerous successful large-scale citizen science projects — such as iNaturalist (www.inaturalist.org), Wildbook^6^ and Coral Watch^7^ in which volunteers collect data — engaging CS for data analysis is relatively less developed. Over the past two decades, online citizen science projects have shown that volunteers can reliably identify species and count individuals from images with minimal training^8^. By using ‘crowdsourcing’ for data analysis (popularly known as ‘the wisdom of crowds’), researchers can massively reduce the amount of specialist time and effort needed to obtain results^9^ while engaging the public in conservation projects. We aim to further explore this approach by applying CS-powered data analysis to surveys of the endangered marine iguana (*Amblyrhynchus cristatus*) — a charismatic well-known species endemic to the Galápagos.

For nine of the 11 subspecies of marine iguana^10^, complete population-size data are lacking and urgently needed, mainly due to monitoring challenges inherent in surveys of this species^11,12^. Given the unprecedented levels of anthropogenic change (e.g., introduced species, climate change, marine pollution) in the Galápagos^13 ^better information is needed. To address this data gap, we employed a recently validated boat-based surveying method that uses drones to collect aerial images of marine iguana colonies^14^. However, this approach generates a large image-based dataset which requires extensive specialist analysis, delaying the acquisition of results and likely reducing the future use of this approach. In seeking a solution, we created the Iguanas from Above project on Zooniverse.org; the largest platform for online citizen science projects^15^. There, we placed a set of images (smaller slices of orthomosaic images created from the raw drone images) from a selection of locations. Each image was shown to a predetermined number of independent volunteers, who were asked to mark all the iguanas found within the image; this task is known as a ‘classification’. Once all images were classified, the resulting raw data were ‘aggregated’ to obtain a consensus result from the individual inputs for each image. Having run three successive phases of our project, we use this as a case study for population-size estimation in wildlife in the Galápagos. This work aims to address the following questions:

1. **In what ways can the raw data of citizen scientists be filtered to improve agreement with the experts?** Here we addressed: (a) does removing inputs from anonymous or infrequent volunteers improve the outcome? And (b) does increasing the number of times an image is independently classified (‘classifications required per image’) increase count accuracy?
2. **Which aggregation method used produces results closest to those of experts?** As we have up to 30 separately entered counts per image (one from each volunteer classification), it is necessary to summarise the outcome on aggregate. Here, we tested traditional measures: median and mode, as well as two clustering approaches that integrate the coordinates of iguanas — as marked by the volunteers in the images — into the analysis.
3. **Which aspects of the images presented to citizen scientists are important when determining the accuracy of the results?** For this, we investigated the phase analysed, the quality of the image in terms of sharpness, blur, lighting, etc., and the number of individuals present in the image as potential factors.
4. **Can citizen scientists accurately identify and count marine iguanas on aerial images?** This is — in essence — our overarching question. We explored whether volunteer counts align with those of ‘experts’ — i.e. professional scientists trained for marine iguana detection in aerial images. Based on previous studies across diverse systems and taxa (e.g^16–19^) we consider CS data accurate if there is > 95% agreement with experts for detecting an object (i.e. correctly identifying the presence or absence of marine iguanas in the image) and > 80% on counting an object (i.e. matching the expert count for the number of marine iguanas in the image). If sufficient agreement is found, we intend to use CS inputs to estimate population size at key colonies of marine iguanas.

## Results

### Volunteer participation insights and filtering

The three phases included in this study were completed in September 2023. At this point, over 10,000 registered volunteers and 3,500 unregistered (anonymous) volunteers (13,988 in total) had made 1,375,201 classifications from 57,838 aerial images. Most volunteers (86%, *n* = 12,099) made less than 50 classifications, yet some classified thousands of images (174 volunteers each classified over 1,000 images). These results agree with those provided by the volunteer survey (supplementary Fig. S1), where most respondents reported having entered 100 or fewer classifications. A small number of ‘super volunteers’ made a significant input: our top 10 contributors represented 18% (n = 244,654) of the total classifications.

Logging-in to classify images is a Zooniverse recommendation but it is optional. Overall, we found that 15% (*n* = 207,942) of our classifications were performed by participants who were not logged-in (3,753 volunteers). Even when users are not logged in, Zooniverse gives them an identification code registered as “not-logged-in + IP number”. A significant amount of data (16%) in the Gold Standard (GS) dataset was generated by anonymous volunteers; when this data was excluded, the accuracy within the ‘iguanas present’ images decreased by 9% in phase 1 and 2% in phase 2 and no reduction in phase 3. Therefore, all classifications — including anonymous inputs — were retained for the analysis.

## Aggregation methods: minimum threshold for accurate CS Detection

**Fig. 1:**
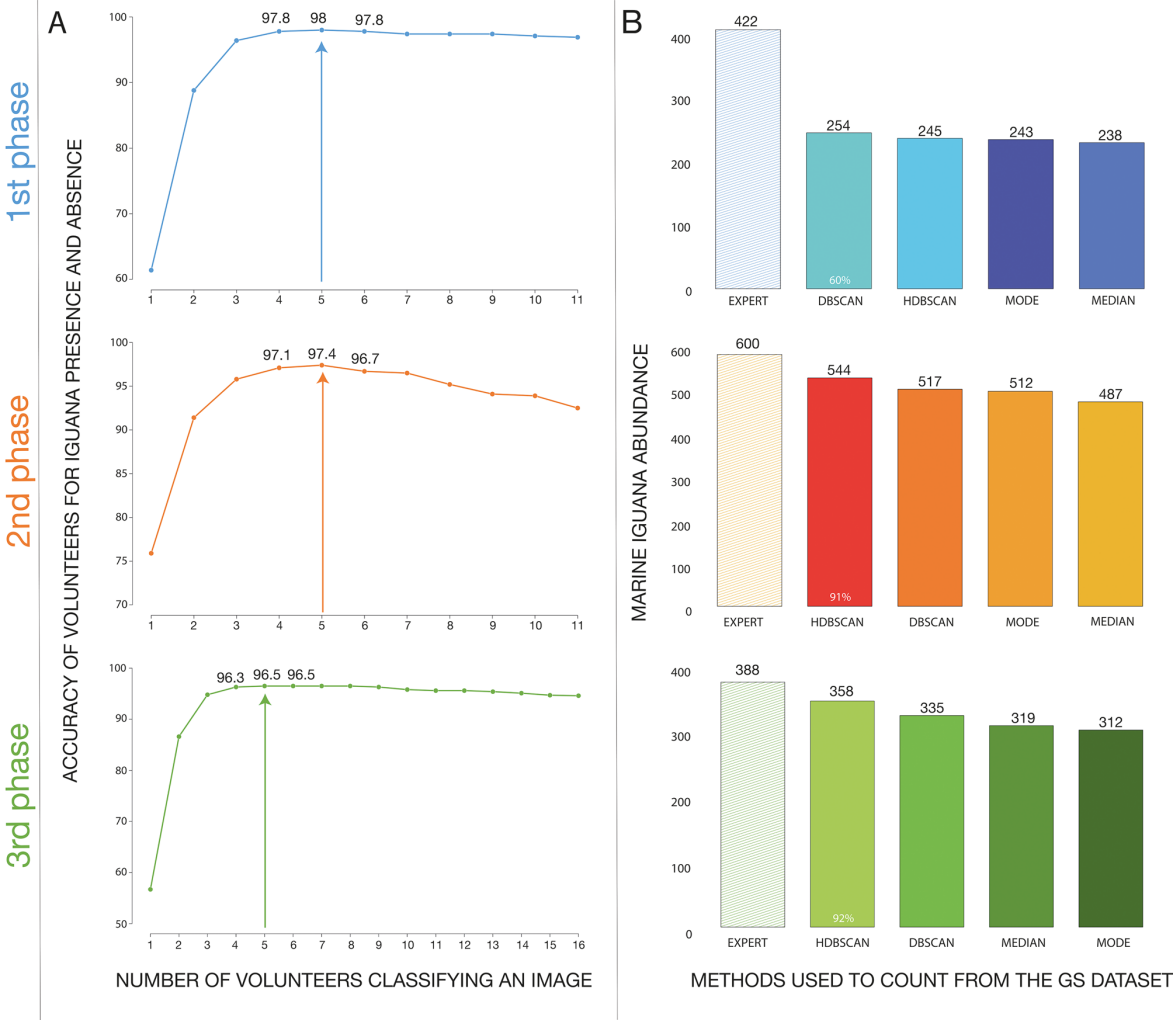
Graphs of results from Tasks 1 and 2. (**A**) Results of the minimum threshold approach for the three analysed phases, and (**B**) Total expert counts compared to total citizen scientist counts across aggregation methods.

Considering all images within the GS dataset (i.e. those with and without iguanas), the highest level of detection accuracy in the volunteer data — when compared to experts — was found when at least 5 or more volunteers (from the 20 or 30 that classified each image) indicated the presence of an iguana. This finding was consistent across all project phases (Fig. 1a). Agreement levels for iguana detection across each phase were: phase 1 (98%; *n* = 2,733); phase 2 (97%; *n* = 456); and phase 3 (97%; *n* = 1,156). Accuracy decreased when the majority vote method was used (phase 1 = 97%; phase 2 = 93%; and phase 3 = 95%, supplementary Fig. S2); therefore, our results suggest that requiring more repeated classifications of an image does not increase CS accuracy in our project. Since the vast majority (90%) of our images were blank (i.e. did not contain an iguana), further analysis was undertaken to assess the effect of this skew on the overall dataset. For this, we applied the minimum threshold of five volunteers as the aggregation method.

When experts did not detect any iguanas in an image, we classified the image as ‘absent’. Using only these ‘absent’ images, we compared the expert results to the CS data. Here, we classified the CS outcome as correct for absence if fewer than five volunteers indicated an iguana present in an image recorded as ‘absent’ by experts. In this, we report an agreement average of 99.6% between experts and CS across the three phases. On the other hand, in images where iguanas were identified by experts (10% of images overall), we classified these as ‘present’. For this, we deemed the CS outcome as correct for presence if five or more volunteers indicated an iguana in an image recorded as ‘present’ by experts. Volunteers classified 68% of these images correctly in phase 1 (*n* = 150), 94% in phase 2 (*n* = 179) and 70% in phase 3 (*n* = 116), meaning that false negatives varied by phase, and were more likely than false positives.

## Aggregation methods: marine iguanas as counted by volunteers

Of the 4,345 images in our GS dataset, experts detected 418 images with iguanas (10%), whilst volunteers found iguanas to be present in 363 images when applying the minimum threshold rule as outlined above. From these 418 images, 349 (84%) had fewer than five individuals counted, indicating the iguanas are typically rare in the images; with just one image having 35 iguanas (the highest counted). For each image, we obtained the aggregated count of the 20/30 volunteers who assessed the image using various metrics for this comparison. When combining GS images from all phases, we found that the volunteers counted the same exact number of iguanas as the experts in 94.3% of the images (median), 94.5% (mode), 94.7% (HDBSCAN clustering analysis) and 93.0% (DBSCAN approach); though this varied by phase from 76 to 97% (see supplementary Table S1). The remaining percentage represent the images where the volunteers under or overcounted iguanas, when compared to the experts. Note that only counts — and not the exact location/identity of the iguanas — was compared.

We found significant differences among the outcomes of the various methods used to count marine iguanas (comparing expert counts and CS data aggregated using the median, mode, and HDBSCAN; *X*^2^_3, 1668_ = 5888.5; *p* < 0.01). Considering only images where iguanas were present, we found that volunteer counts were significantly lower than those of experts when the aggregated counts used both the median (pairwise test; z = 3.34, *p* = 0.0023) and the mode (pairwise test; z = 3.29, *p* = 0.0055). However, expert counts were similar to those obtained using the HDBSCAN approach (pairwise test; z = 2.48, *p* = 0.0632). We did not find significant differences between CS results calculated between the median and the mode (pairwise test; z =−0.248, *p* = 0.9946), the median and HDBSCAN (pairwise test; z = 1.08, *p* = 0.7051), and the mode and HDBSCAN (pairwise test; z = 0.83, *p* = 0.8418).

The logistic regression analysis obtained the highest fit value with the expert counts for the HDBSCAN approach (Nagelkerke R-squared: 0.917), followed by the median (Nagelkerke R-squared: 0.893), and the mode (Nagelkerke R-squared: 0.870 (see supplementary Table S2).

Finally, we compared the sums of all counts across the project and by individual phase. Total expert counts per phase were: 422 iguanas in phase 1, 600 in phase 2, and 388 in phase 3. For total CS counts, the HDBSCAN obtained the highest agreements in phase 1 (58%), phase 2 (91%) and phase 3 (92%). The median and the mode followed these results. Overall, the overcounting percentages for these methods were at most 5% (when excluding DBSCAN, which produced suboptimal results) (Table 1; Fig. 1b).

**Table 1:** Marine iguanas counted in the gold standard (GS) dataset. Number of iguanas counted by the experts and the CS for all the aggregating methods tested. Iguanas undercounted are the number of individuals that CS counted under expert counts, and iguanas overcounted are the number of individuals that CS counted over the expert counts. Bold values represent the best results of the aggregation methods (excluding DBSCAN) by phase and column category.

**Expert counts**	**Aggregation Method**	**Total** **CS counts**			**Iguanas** **undercounted**			**Iguanas** **overcounted**	
		**Iguanas**	**%**		**Iguanas**	**%**		**Iguanas**	**%**
1410 (All phases)	Median	1044		74	420		30	54	**4**
	Mode (max)	1067		76	420		30	77	5
	HDBSCAN	1147		**81**	333		**24**	70	5
	DBSCAN	1106		78	443		31	139	10
422 (1st phase)	Median	238		56	196		46	12	**3**
	Mode (max)	243		58	210		50	31	7
	HDBSCAN	245		**58**	192		**45**	15	4
	DBSCAN	254		60	203		48	35	8
600 (2nd phase)	Median	487		82	138		23	25	**4**
	Mode (max)	512		85	117		20	29	5
	HDBSCAN	544		**91**	83		**14**	27	5
	DBSCAN	517		86	139		23	56	9
388 (3rd phase)	Median	319		82	86		22	17	**4**
	Mode (max)	312		80	93		24	17	**4**
	HDBSCAN	358		**92**	58		**15**	28	7
	DBSCAN	335		86	101		26	48	12

## Factors affecting volunteer classifications

**Fig. 2:**
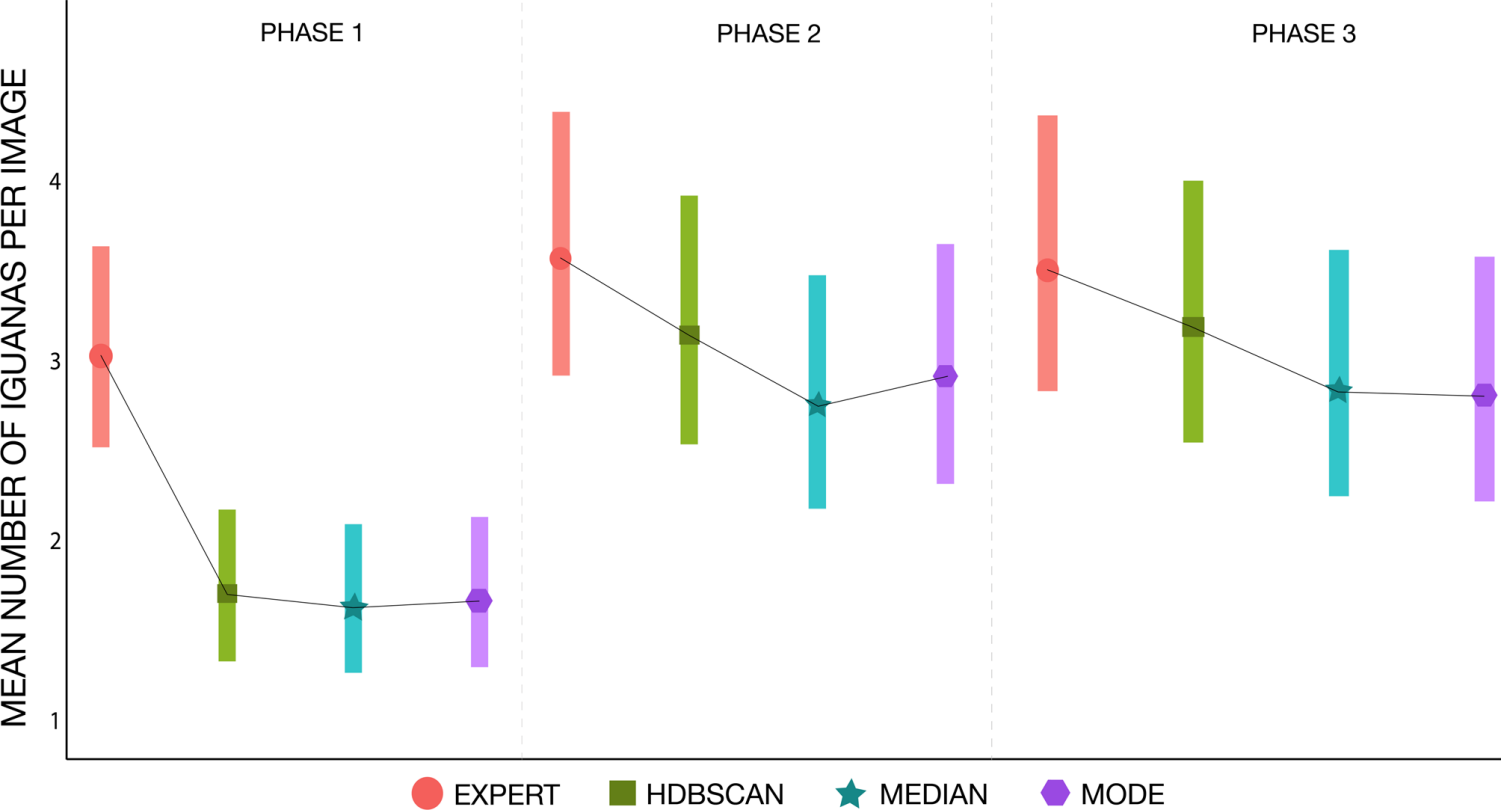
Plot of the generalised linear model (analysed by phase independently) when all the methods used to count marine iguanas from the Gold Standard images were compared. The black lines join the means of all methods, and the bars represent each method’s data variation (confidence intervals).

We investigated whether the factors: phase of the project; quality of the image (classified as “good” or “bad” by the experts); and number of iguanas present in the image (in three categories of low, medium, or high; see methods for further details) potentially affected the volunteer counts. When we added the factor “phase” to the model [glm(counts ~ method + phase, family = quasipoisson)], we found significant differences among the methods (*X*^2^_3_, _1668_ = 5,888.5; *p* < 0.01). Specifically, expert counts were significantly higher than CS-counts as calculated with the median (pairwise test; z = 3.65, *p* = 0.0138) and the mode (pairwise test; z = 3.40, *p* = 0.0326), but not with the HDBSCAN (pairwise test; z = 2.56, *p* = 0.3013). When analysing each phase independently, however, the results revealed discrepancies between phase 1 and the other two. In phase 1, experts significantly count more iguanas than any of the CS methods [median (pairwise test; z = 3.92, *p* = 0.0005), mode (pairwise test; z = 3.80, *p* < 0.0008) and HDBSCAN (pairwise test; z = 3.69, *p* = 0.0013)], whilst phase 2 and phase 3 show no significant differences among counts (*X*^2^_3_, _644_ = 2,777.3; *p* = 0.36, *X*^2^_3_, _440_ = 1,875.0; *p* = 0.48, respectively; Fig. 2).

When we added the factor ‘image quality’ to the model [glm(counts ~ method + quality, family = quasipoisson)], we found significant differences among the methods (*X*^2^_3_, _1668_ = 5,888.5; *p* < 0.01) but not between the quality (*X*^2^_1_, _1667 _= 5,885.1; *p* = 0.42). Expert counts were significantly higher than the CS counts, both in images with good and bad quality, when using the median (pairwise test; z = 3.53, *p* = 0.0099) and the mode (pairwise test; z = 3.29, *p* = 0.0225), but not significantly different with the HDBSCAN (pairwise test; z. ratio = 2.48, *p* = 0.2056). Interestingly, we found different results when we investigated the influence of image quality on CS-counts for phase 1 when analysed independently. Results show that on images with poor quality, all the counting methods obtained higher numbers than on images with good quality (*X*^2^_1_, _575 _= 1,461.4; *p* < 0.01; see supplementary Fig. S3). Regardless of image quality, the expert counts were significantly higher than all the CS methods: median (pairwise test; z = 3.95, *p* = 0.0020), mode (pairwise test; z = 3.84, *p* = 0.0032) and HDBSCAN (pairwise test; z = 3.72, *p* = 0.0049). However, when analysed separately, differences in phase 2 are no longer significant between methods or quality (*X*^2^_3_, _644_ = 2,277.3; *p* = 0.36, *X*^2^_1_, _643_ = 2,276.9; *p* = 0.79, respectively). This was also true for phase 3 across methods (*X*^3^_23_, 440 = 1,875.0; *p* = 0.48) and image quality (*X*^2^_1_, _439_ = 1,869.0; *p* = 0.30) (Fig. S3). Apparently, phase 1 greatly influenced the results of the model, which is likely explained by the poor image quality that characterised this particular dataset.

Finally, when the factor ‘number of iguanas present in the image’ was added to the model [glm(counts ~ method + quantity, family = quasipoisson)], we found that expert counts were significantly higher than the CS counts across all methods used in all categories of abundance (see Methods): median (pairwise test; z = 4.67, *p* = 0.0002), mode (pairwise test; z = 4.36, *p* = 0.0008) and HDBSCAN (pairwise test; z = 3.29, *p* = 0.0462). This result again differed when the analysis was performed independently for each phase. In phase 1, significant differences between the expert and CS data from various metrics remain regardless of the number of iguanas present: median (pairwise test; z = 4.59, *p* = 0.0003), mode (pairwise test; z = 4.45, *p* = 0.000) and HDBSCAN (pairwise test; z = 4.40, *p* = 0.007). Conversely — as with the phase and image quality analyses — there were no significant differences between the expert and CS counts in all categories of abundance for both phase 2 (*X*^2^_3_, _1820_ = 6,513.8; *p* = 0.08) and phase 3 (*X*^2^_3_, _4620_ = 8,236.4; *p* = 0.24) (see supplementary Fig. S4). In conclusion, volunteers can count accurately even when the number of iguanas on an image is high, but where image quality is concurrently poor — a scenario more frequently encountered in phase 1 — CS accuracy significantly decreases.

## Inputs from the volunteer survey

We received 110 inputs in our volunteer survey. Regarding perceived difficulty in classifying images, the key issues reported were image blur, poor camera focus, and similarity between the substrate (typically dark rocks) and the iguanas (supplementary Fig. S5a). Very few volunteers noted that (too) many iguanas was a problematic feature of the images (supplementary Fig. S5b), though these images were relatively rare within the dataset and thus it is not clear how significant this issue may be in colonies with higher abundance. In terms of engagement, a lack of iguanas in the images (i.e. ‘blank’ images) was the most important factor in decreasing motivation, followed by poor image quality, substrate and object similarity, and task repetitiveness (supplementary Fig. S6).

## Discussion

### Which aggregation method used produces results closest to those of experts?

Remarkably, we found that the frequently used simple plurality algorithm (‘majority vote’^9,20^) did not produce the most reliable data for detecting presence or absence of iguanas. Instead, we found that the answer selected by five volunteers or more (from 20 or 30 total classifications) — which we refer to as the minimum threshold — was the most likely to be correct. This was unexpected since some previous work has shown that accuracy increased asymptotically with the number of classifications per image^18^. However, other projects hosted by Zooniverse have also found similar outcomes^21–23^. We suspect that for some challenging images, highly skilled volunteers were required to identify the iguanas and that these volunteers were relatively rare. Therefore, if we applied the majority vote rule, we would require 11 of 20 volunteers who viewed each image to identify the iguana, whereas using a minimum threshold of five would require just five of 20, giving relatively more weight to the highly skilled ones. Each project might, therefore, need to determine its own minimum threshold for classification aggregation, especially when the majority rule does not produce the required accuracy. If this alternative method still doesn’t achieve the expected accuracy, adding more classifications might increase the probability of finding those skilled volunteers. However, this is — of course — a trade-off, since more classifications entails a longer data analysis phase.

In images where experts found no iguanas (‘absent’ or ‘blanks’), CS results were in 99.6% agreement; thus, our results show a very low instance of false positives (i.e. volunteers mistakenly indicating iguana presence). This tendency can vary among projects; for instance, in Snapshot Serengeti, volunteers rarely produced false positives^24^. However, in the “Año Nuevo Island Animal Count”, volunteers were more likely to detect non-existent individuals than to miss existing ones^25^. Although the overall 97% accuracy for the presence/absence task is superficially impressive, further analysis is warranted. This is because most of our images were blank — i.e. did not contain an iguana, which skews the data. The majority of this 3% error stems from analysis of images where iguanas were present, which was only 10% of the overall images. Within this ‘presence’ data subset, the error rate — so-called ‘false negatives’ — is much higher (from 6–32%); meaning that in our case, volunteers were more likely to miss marine iguanas on the images, leading to undercounting.

Regarding counting iguanas, we tested various metrics for summarising (‘aggregating’) multiple CS inputs to see which produced the most similar results against the expert counts. We found no significant differences among the methods used, although the HDBSCAN obtained the highest accuracies overall in counts for all phases and gave the statistical best fit to the expert counts. In contrast to traditional metrics like median and mode, this approach considers the spatial marks made by volunteers on the images when locating the iguanas. These marks are grouped into clusters, with each representing an individual animal if identified by a minimum number of volunteers — in this case five, using the minimum threshold rule. This additional spatial information may explain the superior performance.

Moreover, the coordinates of clusters could prove useful for addressing other questions, such as those related to behaviour or habitat use^25^and providing training data for Machine Learning approaches^26–28^. We tested both HDBSCAN and the related approach DBSCAN. We found that the former was not only easier to use — because it required fewer parameters to be set — but also obtained better clustering outcomes (see supplementary Text S1 for details). Employing the HDBSCAN clustering analysis, we found that total CS-counts for phases 2 and 3 were 91% and 92% accurate when compared to experts. When the CS-counts per image were analysed statistically by phase, we found they were not significantly different to those of the experts. However, we note that the difference between the results from HDBSCAN and the median was relatively minor.

### In what ways can the results from the raw CS dataset be filtered to improve agreement between citizen scientists and experts?

We investigated the impact of filtering input from anonymous (i.e. users not logged into the platform) and ‘inexperienced’ volunteers (i.e. those with 10 or fewer classifications) from the dataset. We found that anonymous users contributed around 15% of our total classifications, while inexperienced volunteers represented 76% of all the participants and contributed with 13,200 classifications (9%). Eliminating input from such participants has been helpful in some citizen science projects^29 ^however — as noted by Swanson et al.^18^ — when having multiple independent classifications per image (crowdsourcing), eliminating classifications often means discarding significant amounts of volunteer effort and potentially valuable information. In our case, removing anonymous inputs significantly reduced the overall number of classifications, resulting in reduced accuracy. Worth noting was that some anonymous volunteers undertook several thousands of classifications (up to 11,606), indicating that being logged-in — and thus potentially receiving recognition for inputs — is not necessarily a reliable indication of engagement with a project.

## Which aspects of the images presented to citizen scientists are important when considering their accuracy?

Particularly in phase 1, poor image quality significantly affected the results — this was most obvious in images collected from El Miedo on Santa Fe Island, which was also coincidentally the colony with the highest density and smallest iguanas within that phase and is thus the primary reason for the higher values of iguana undercounting here. This colony was surveyed as part of our pilot phase (January 2020), where our drone protocols were not optimal in terms of altitude and image overlap, producing comparably lower-quality images. Moreover, in this initial phase, we added a watermark to each image on Zooniverse, which negatively influenced the visibility of objects. Our image collection procedure has since significantly improved; this impact is evidenced by the results obtained in phases 2 and 3. For this reason, the two later phases are more representative of the approach and thus are used here to validate the method.

In addition to image quality, the general predominance of ‘blank’ images within the dataset was also an issue. This scarcity of images with iguanas undoubtedly reduced volunteer opportunities to ‘learn by doing’. This issue is common among projects where focal objects are rare, too small, or too similar to the background^25,30^ and may have contributed to the false negative error rate, particularly in phase 1 where the density of iguanas on one of the surveyed islands — San Cristobal — is extremely low^31^. Undercounting is also common when several individuals are present in the image — generally, the more individuals per image, the lower the agreement between CS and the experts^24,32^. However, our statistical analyses showed that volunteers can also count accurately when several iguanas are present, except in phase 1, where image quality was a compounding factor.

### Can citizen scientists accurately identify and count marine iguanas on aerial images?

To address this question, we must consider both the participation rate of the volunteers — to assess whether analysing a dataset within a reasonable timeframe is likely — and the accuracy of the volunteer-generated data. In our cumulative participation curve, we experienced a daily classification rate of 200–9800; the higher-end numbers being in response to promotional work, in keeping with results from other projects^16,33^. Crowdsourcing undoubtedly offers the opportunity to analyse large datasets with relatively little expert input, but without active promotion and engagement, it can take considerably longer to complete the analysis^21^. Each phase of our project took between 5 and 14 months to complete. This timeframe was influenced by the size of the phase dataset, with an overall average of almost 1900 images being fully analysed each month (phase 1: 2031; phase 2: 1819; phase 3: 1740). This length of time was manageable for our purposes, and there are straightforward ways to speed up the process via promotion if time is pressing.

After applying the best-performing aggregation method to the CS data and omitting suboptimal data from the project’s pilot phase, we found that CS-counts were 91% and 92% accurate when compared to those of experts. This meets the criteria we defined as ‘accurate’ in terms of counts, and thus, we find the approach suitable for counting marine iguanas. Although expert counts in phases 2 and 3 are not significantly higher than those from the CS data, a tendency for volunteers to undercount is still evident. This indicates the need to calculate and apply a correction factor if CS inputs are used to estimate the population size.

Since CS projects rely entirely on volunteer inputs, it is important to consider the volunteer experience and factors motivating participants. A study by Aceves-Bueno et al.^34^ found that participants who received economic recognition outperformed those who did not. Whilst this type of ‘reward’ may be helpful, our experience here indicates that recognition for work undertaken is not a prerequisite for involvement. Our finding matches those projects where many participating volunteers remained anonymous (unregistered)^32,35^. Volunteers in these types of projects seem not to expect external rewards but may rather be motivated by the intrinsic desire to contribute to science/conservation, engage with researchers, and be involved in scientific discussion^36^. Another pattern noted in previous work, confirmed in our project, is that most participants contribute few classifications, while few volunteers contribute many^21,37^. In our case, we found several volunteers who classified thousands of images, including one who looked at all images within each phase. This finding was confirmed by the volunteers’ replies to our survey, where most users responding estimated themselves to have contributed up to 100 classifications. This finding makes a strong case for using multiple independent classifications for each image; this allows a dataset to be rapidly analysed even when each user’s contribution is small.

It is also important to consider factors that reduce volunteer motivation; in our case, the infrequency of marine iguanas in the images seems to have been important, as reported by the volunteers (supplementary Fig. S6). Interestingly, other researchers have found that such ‘blank’ images motivated the volunteers to keep looking for the target, and when blanks were removed, their participation time decreased^38^; though it is worth noting that this study did not explicitly test the number of classifications made in relation to the proportion of blank images. In our dataset, where 90% of our images were blank, volunteers contributing only a few classifications may not have seen any iguanas and may have also spent a large amount of time attempting to distinguish these objects from a visually similar substrate.

### Consideration of online citizen science projects

Image-based datasets for wildlife monitoring are increasingly used, in great part due to technological advances that have made devices — such as camera traps and drones — more affordable and more suitable for such work. However, although these approaches can reduce survey time, the effort required for image analysis can constitute a considerable burden for projects with limited resources. Online citizen science projects — which involve the collaboration of the general public to analyse images remotely — are helping to resolve this issue, offering key advantages such as increased cost-effectiveness and a significant reduction in workload.

Despite the historical debate regarding the accuracy of citizen science-generated data^34 ^online CS is now recognized as an important approach for large-scale ecological research^29,30^. Multiple studies have found that researchers can obtain accurate volunteer data by properly aggregating multiple independent responses for one subject (e.g. an image or audio file)^18,22,26,37^. In parallel, researchers may also use CS to engage and educate the public on themes related to science and conservation^17^. Studies have shown that citizen scientists participating in nature-focused projects tend to develop positive environmental attitudes^39^. Therefore, involving the public may benefit the project and help the scientific community, aid in public engagement and education, and increase interest in topics related to biodiversity and the environment.

One big draw of the CS approach is that by crowdsourcing, researchers can meet their aims in less time and/or expand their aims past what would be possible using more traditional approaches^27,33^. A review across 17 CS projects estimated that the CS approach allowed analysis to be completed on average within 2.4 years per project, as opposed to the 37 years estimated if experts classified the data^8^.

Large-scale monitoring in the Galápagos is logistically extremely challenging and is, therefore, rare^40,41^. For most of the species, the majority of studies focus on just a few colonies^42,43^. For the marine iguana, surveying the whole range of the species is only realistic when new approaches — such as drone-based surveying — are applied. Still, analysis of the large datasets generated remains a significant obstacle to the completion of this work. Here, we confirm that aerial images have the potential to provide reliable data from volunteers with little training, indicating a reasonable approach to alleviate the analysis bottleneck.

Moreover, the images can address numerous questions about other taxa and the environment. Apart from the tasks related to the marine iguanas, we also asked the volunteers to classify other species and detect plastic objects; this is data we could easily collect alongside our tasks, which will be made available to other researchers as a contribution to their work. Combining an image-based method for surveying remote areas with crowdsourced analysis via online CS can be a valuable approach in collecting and analysing large datasets.

### Conclusion and further work

Our results validate the use of the citizen science approach to accurately identify and count marine iguanas from aerial images. However, there is a tendency to undercount the number of iguanas. Our next step is to continue our analysis, which includes images from the most populated colonies, to identify a correction factor that will allow CS inputs to be used for accurate population-size estimates of marine iguanas. This is possible because experts have already validated the counting of iguanas from aerial imagery against traditional ground-based approaches^14^. This work is an essential contribution to our overall goal of addressing the population-size data-gap that currently hampers the effective conservation of this species^12^.

In future work, we expect to use our images to analyse reproduction dynamics, and potentially habitat characteristics, using a CS approach. We are interested to see whether volunteers can reliably identify certain aspects of the colonies, such as the presence of leks and males with breeding colouration; these data will be helpful to address the dearth of information regarding marine iguana breeding activity across the archipelago.

Our next major goal is to use machine learning (ML) to analyse drone imagery. As with several other projects^26–28,44^ CS-input is being used to train Artificial Intelligence for pattern recognition and minimize training time for the computers. We also expect to use ML to filter data, enabling us to remove blank images from our CS datasets and focus this human effort on the most important images. We envisage that this will improve volunteer participation and decrease the running time of the online project. By combining CS with ML, we aim to create a semi-automated pipeline capable of finding and counting marine iguanas and other biologically relevant objects in drone images, significantly reducing the effort needed to undertake such work.

### Methods

#### Building the citizen science project

**Fig. 3:**
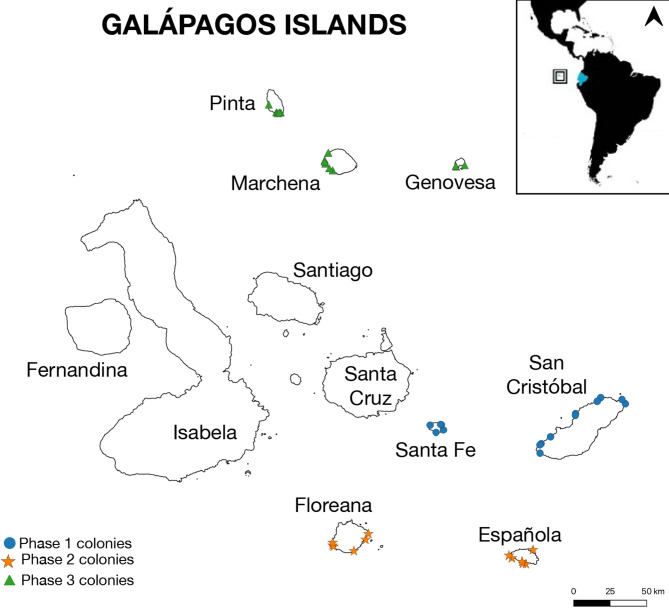
Map of the Galápagos Islands. The coloured points represent all the colonies included in this study. The three phases analysed belong to seven major islands. The square in the upper right shows the geographic location of the Archipelago, ~ 1,000 km from the coast of continental Ecuador.

We collected the aerial images used for this project using commercial drones (DJI Mavic 2 Pro), flown from land and boats along the rocky coastline of several marine iguana colonies in the Galápagos Archipelago during three successive field seasons from 2020 to 2022 (Fig. 3). With the images, we created orthomosaics (2D-georeferenced maps) using the software Agisoft Metashape; for full details on image collection and analysis, see Varela-Jaramillo et al.^14^. We ‘sliced’ the orthomosaics using Adobe Photoshop to create individual images of 1,000 × 1,000 pixels on average (resulting image size: up to 1 MB). We did this in an attempt to standardize the size of the individual iguanas in the images to aid recognition, as this depends on the height at which we flew the drones (20–30 m altitude), which varied in response to the body size of each subspecies of marine iguanas monitored.

**Fig. 4:**
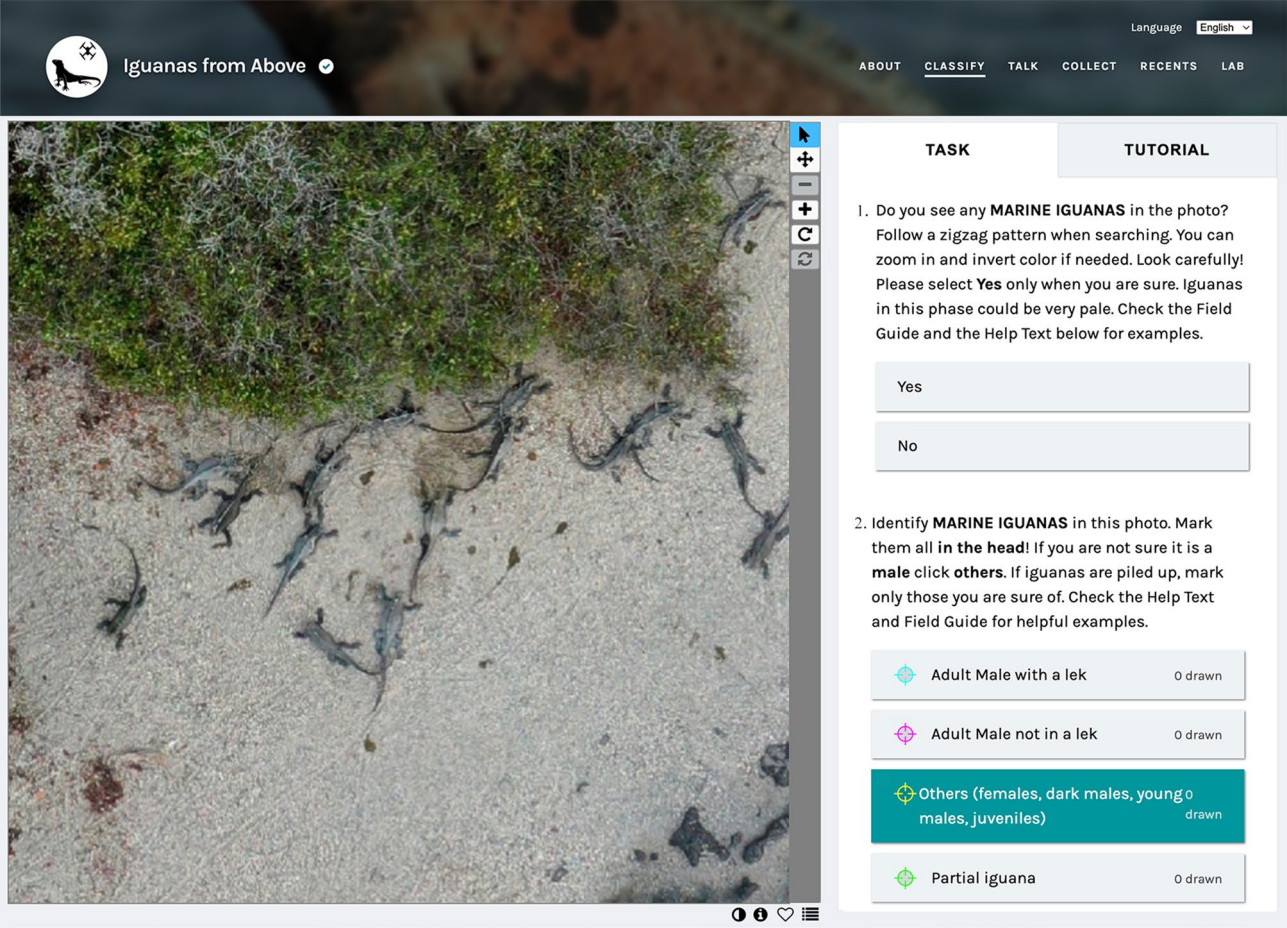
Iguanas from Above citizen science web portal in Zooniverse.org. An example tile with the two tasks presented to citizen scientists. Task 1 involves identifying (detecting) marine iguanas present or absent in the image, and Task 2 refers to the number (counting) of individuals present in the image.

We created our project on the Zooniverse platform in English, with translations available in Spanish, French, and German. Our workflow for the project requires volunteers to complete three tasks for each image classification (Fig. 4): (1) identify presence or absence of marine iguanas in the image; (2) mark individuals of marine iguanas, distinguishing adult males and reproductive groups (leks) from ‘others’ (females, sub-adult males, and juveniles) when possible. The category ‘partial iguana’ was an addition made to avoid double-counting of occasional individuals that were bisected at the edge of the image during slicing (this was explicitly explained to the volunteers via the training on Zooniverse); and (3) identify and count individuals of cohabiting species, which included sea lions (*Zalophus wollebaeki*), crabs (*Grapsus grapsus*), Green Turtles (*Chelonia mydas*), sea birds, plants, and algae (various species), as well as plastic objects (bottles and fishing gear). We provide a tutorial and a ‘Field Guide’ for species identification. A message board for discussion is enabled where volunteers interact with researchers and each other regarding image classification; this also provides a space for ongoing discussion on Galápagos wildlife and conservation matters.

To date, we have launched five phases of the Zooniverse project, each following an annual field trip undertaken between 2020 and 2023, during which we surveyed all islands within the mating season of the marine iguana, when the iguanas aggregate in breeding territories. For this study, we analysed the first three complete phases. Analysis of phase 1 began in August 2020 and comprised 24,373 images from San Cristobal and Santa Fe islands surveyed as a pilot project in January 2020. The three subspecies covered have medium to large body sizes^10^ and relatively low densities. Phase 2 — launched in February 2022 — included 9,097 images from Española and Floreana islands, collected in January 2021. This phase featured the more colourful and abundant “Christmas iguana”, which has a medium to large body size and is renowned for the turquoise colouration displayed by males during the mating season. Phase 3 was launched in July 2022 with 24,368 images from the northern islands of Genovesa, Marchena, and Pinta, surveyed in December 2021. Marine iguanas on these islands are rare, small-bodied, highly cryptic against the rocky substrate, and males appear to lack the colouration seen on other islands in the pairing season. Two sites from Phase 2 were also included here.

We circulated each image among a predefined number of independent volunteers, whose input is referred to as a classification. We required 20 classifications per image for phases 1 and 2, and 30 classifications for phase 3. The extra 10 classifications in the latter phase were intended to test whether more classifications per image would improve CS accuracy.

To increase volunteer participation, we undertook promotional activities including press releases, newsletters, inviting inputs from schools, universities and companies via webinars, social media and blog posts, as well as participating in the Citizen Science Month event promoted by SciStarter (https://scistarter.org/iguanas-from-above) and being a featured Zooniverse project. We also ran a competition to award the best classifiers in phase 2 to motivate the volunteers.

### Building the Gold-Standard dataset for CS-data analyses

**Fig. 5:**
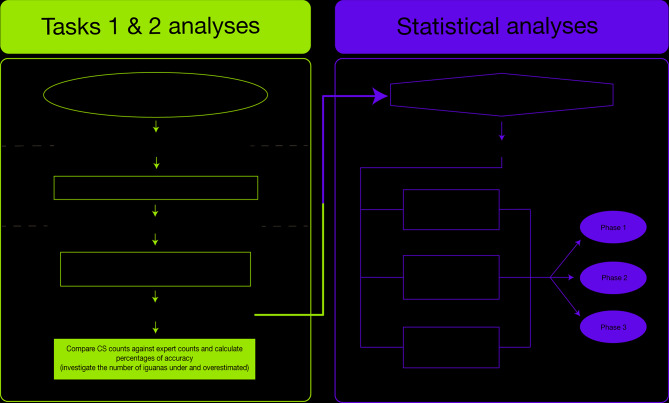
Workflow used to analyse citizen science data obtained for our project. (**A**) Gold Standard dataset creation, (**B**) aggregating citizen scientists’ data for Task 1, (**C**) aggregating citizen scientists’ data for Task 2 and comparing with expert data, and (**D**) statistically exploring potential factors influencing citizen scientists’ accuracy.

We used RStudio version 2023.09.1 + 494^45^ and Python version 3.10.13^46^ with the library Pandas version 2.1.2^47^ for data frame management, ensuing analyses and plotting of results. We downloaded CS-classifications from the Zooniverse platform and used the Panoptes Aggregation python package (See Code Availability for Panoptes script) to extract and summarise (‘aggregate’) data from Task 1 (marine iguana presence/absence – question-type data) and to extract data from Task 2 (marine iguana counts – point-type data). We randomly selected around 5–10% of the images per phase as a Gold-Standard (GS) dataset; these images were chosen to cover a range of challenges, including a variety of iguana body sizes and colouration, both high- and low-density colonies, and from various fieldwork years (since quality of images improved throughout the project). This included 4,345 images from 30 colonies selected from 7 major islands: 2,733 images from phase 1, 456 images from phase 2 and 1,156 images from phase 3. Three people from the research team (henceforth referred to as ‘experts’) analysed the GS datasets and generated consensus results (henceforth called ‘expert’ data) for presence/absence, number of iguanas in the image, and a judgement on image quality (simply “good” or “bad”, Fig. 5a). Image quality judgement was based on the sharpness of camera focus, light levels within the image, image blur due to camera movement, image ‘smear’ due to mosaicking artefacts, and complexity of the substrate on which the animals occur. The expert consensus count was obtained using a two-step process. First, two experts analysed the images independently, then compared results. In cases of disagreement, images were discussed in person to attempt an agreement over whether an object in question was an iguana or not. If an agreement was not reached, the images were then analysed by a third expert. Of the ‘uncertain’ iguanas, only those that were then confirmed by the third expert were accepted as correct. From 4,345 GS images, experts disagreed in 33 images (0.8%), in most cases this was due to an “iguana” marked by only one expert, and therefore these uncertain iguanas were not included in the consensus. We then compared this ‘expert data’ to the CS data for each phase, and for all images together.

### Marine iguana presence or absence analyses

In the online task, each volunteer is asked whether marine iguanas are present (‘yes’) or absent (‘no’) in the image; therefore, for each image in our dataset, we obtained 20 (phase 1 & 2) or 30 (phase 3) answers. A commonly used approach for analysing results where multiple volunteers give input is the ‘simple plurality algorithm’ (also known as ‘majority vote’)^9^. This is where the answer selected by 50% plus one of the volunteers for any given task is accepted for this image. For example, in our case, where 20 volunteers classified an image and 11 or more selected ‘yes’ to indicate presence of an iguana, the result would be ‘iguana(s) present’. We sought a minimum agreement level of > 95% with the expert classifications. We were interested in testing the performance of the ‘majority vote’ approach, while specifically seeking to test whether a smaller number of volunteers could also give accurate results. For this purpose, we calculated the agreement between CS and expert 11 times for each image, determining an agreement level for the case where one of 20 volunteers selected ‘yes’, then two of 20, and so on up to 11 of 20. The aim was to find a ‘minimum threshold’ for the smallest number of volunteers indicating iguana presence in (95%+) agreement with the experts (Fig. 5b; see Code Availability for Minimum Threshold Search script).

We also analysed if removing anonymous (i.e. users not logged into the platform) inputs increased volunteer accuracy in identifying marine iguanas to explore whether project loyalty and volunteer experience might affect the reliability of inputs. Likewise, we tested how removing inputs from volunteers with 10 or fewer classifications affected accuracy, since we hypothesised that these infrequent participants have less experience at the task and thus may be less skilled.

### Marine iguana count analyses

We analysed whether, after selecting yes for marine iguana presence, volunteers were able to detect all the marine iguanas present in the image. In this task, volunteers added marks to the image where they detected the iguanas (Fig. 4). To analyse these outputs, we first selected all images where iguanas were present (obtained using the ‘minimum threshold’ rule) and aggregated the volunteer counts for each (Fig. 5c). We calculated two statistical metrics to aggregate these counts: the median and the mode. The median searches for the value in the middle of an ordered data sample, while the mode seeks the most frequently repeated value within the sample, thus eliminating the outlier effect.

Additionally, we tested the Density-Based Spatial Clustering of Applications with Noise algorithm (DBSCAN) and the Hierarchical Density-Based Spatial Clustering of Applications with Noise algorithm (HDBSCAN) to collate the volunteer annotation marks into spatial clusters of points within the image. The number of clusters then represents the number of iguanas present in the image (see supplementary Fig. S7 and Text S1 for further details on aggregating methods and Code Availability for Clustering script^48^). We excluded data for the ‘partial iguana’ category (i.e. those bisected by the image) since they were very rare and thus, we lack sufficient data for analysis (e.g. only 11 cases in phase 1). From these methods, we investigated volunteer accuracy by comparing counts between experts and CS for each image. We summed all GS aggregated counts by the method used, and compared these to the expert counts, calculating a percentage of agreement (‘accuracy’), as well as the number of iguanas missed (undercounted) and over-counted. We also looked for: the number of images where volunteer counts were in 100% agreement with expert counts; where volunteers counted fewer iguanas than the experts; and where CS counts were higher than experts.

### Statistical analyses regarding volunteer accuracy when counting marine iguanas

We statistically explored — using generalised linear models with a quasi-poisson error matrix and Chi-square tests — how similar the counts in the GS images were between the experts and the volunteers (comparing median, mode, and HDBSCAN). DBSCAN was excluded here due to poor initial results that were related to higher percentages of overcounting. Subsequently, we performed pairwise comparisons using the Estimated Marginal Means R package (emmeans)^49^ to test which of the volunteer counting methods produced results most similar to the expert counts (Fig. 5d). The emmeans package allows pairwise comparisons from generalised linear models on non-normally distributed data, such as the counts obtained in this study. Further, a logistic regression was performed to find the CS aggregation method which best fits the expert data.

We also investigated the effect of three factors on volunteer counts using the same statistical analyses. These were: phase analysed; image quality (see GS methods for criteria); and the number of iguanas present on the image (three categories were defined based on data distribution: Low: 1–5, medium: 6–10, and high: >10 marine iguanas; supplementary Fig. S8; see Code Availability for Statistical Comparisons script).

### Citizen scientists’ experiences

To better understand the volunteers’ experiences, we undertook a short online survey. These included questions about the number of images classified, factors affecting their motivation to participate, and differences they perceived between the phases (see supplementary Text S2 for details). These results were explored to better understand the relationship between volunteer perceptions and our results and were also used to improve our ongoing CS work.

## Electronic supplementary material

Below is the link to the electronic supplementary material.

Supplementary Material 1

## Data Availability

The raw inputs from the volunteer-based (anonymised) classifications of the Gold Standard images and the resulting aggregated dataset for presence/absence and marine iguana counts per image are available in FigShare (https://doi.org/10.6084/m9.figshare.25196306).

## Declarations

### **Accordance**

All methods were performed following Zooniverse.org guidelines and regulations. Zooniverse best practices can be found here: https://help.zooniverse.org/best-practices/.
