# Supplementary material for: Citizen scientists reliably count endangered Galápagos marine iguanas from drone images
Source: Sci Rep. 2025 Jul 24;15:26884. doi: 10.1038/s41598-025-08381-9 (PMC12289890; doi:10.1038/s41598-025-08381-9)
Supplement: Supplementary file 1 — Supplementary Material 1 [file 41598_2025_8381_MOESM1_ESM.docx]

**SUPPLEMENTARY MATERIAL**

**Iguanas from above: Citizen scientists reliably count endangered Galápagos marine iguanas from drone images**

**Authors:** Andrea Varela-Jaramillo, Christian Winkelmann, Andrés Mármol-Guijarro, Juan M. Guayasamin, Gonzalo Rivas-Torres, Sebastian Steinfartz, Amy MacLeod

**Correspondence:** [amy.macleod@uni-leipzig.de](mailto:amy.macleod@uni-leipzig.de)

1. **Supplementary Figures**

**Fig. S1.** Distribution graph of the number of classifications done by all the citizen scientists who participated in our three phases (A) and the estimated number of classifications undertaken by 110 volunteers who replied to our survey (B).


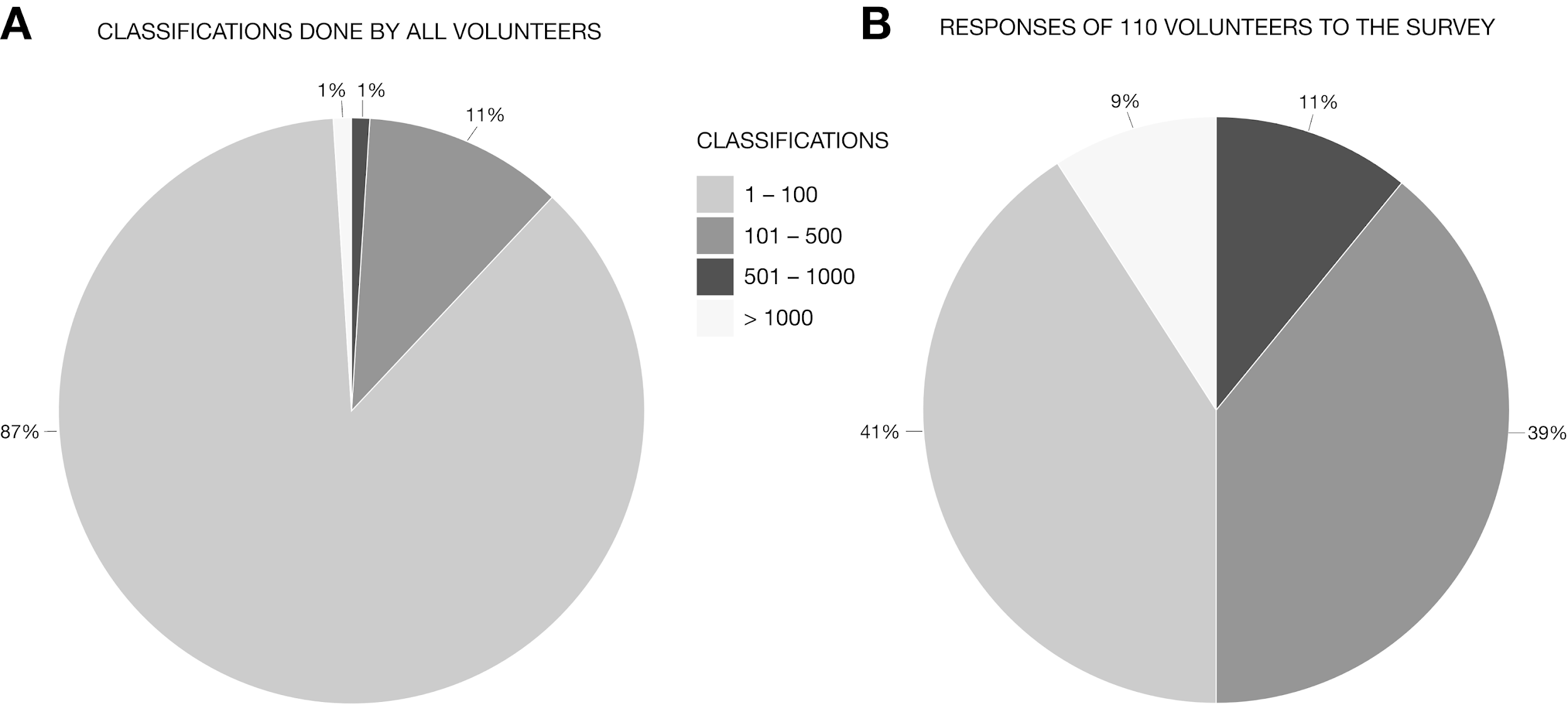


**Fig. S2.** Results regarding CS accuracy in the GS dataset (on images with iguanas present and absent) when using the majority vote approach to assess if an answer was correct or incorrect after comparing it against the expert answer.


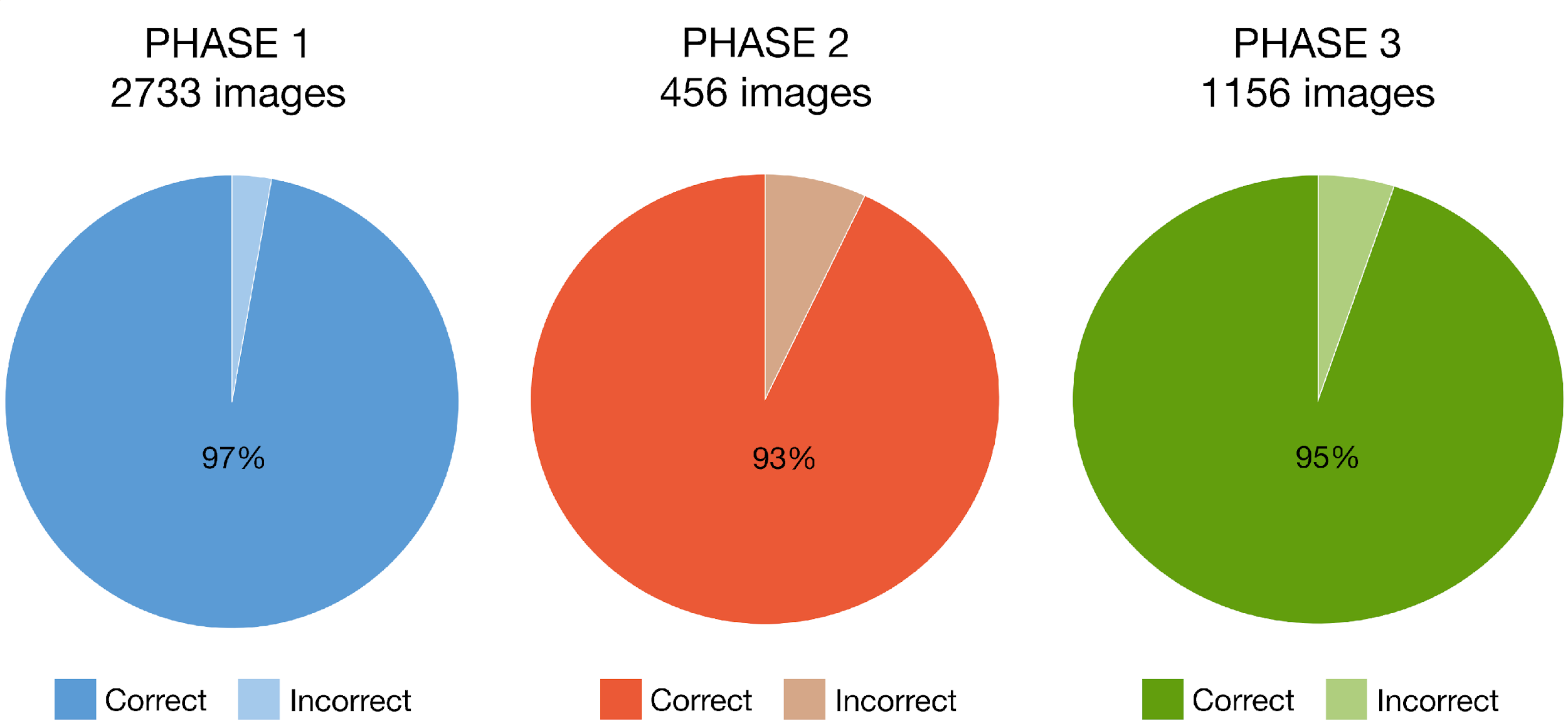


**Fig. S3.** Plots of the generalized linear models are presented independently by phase, with the factor ‘quality of the image’ added into the analysis, assessing differences amongst methods used to count marine iguanas from the GS dataset.


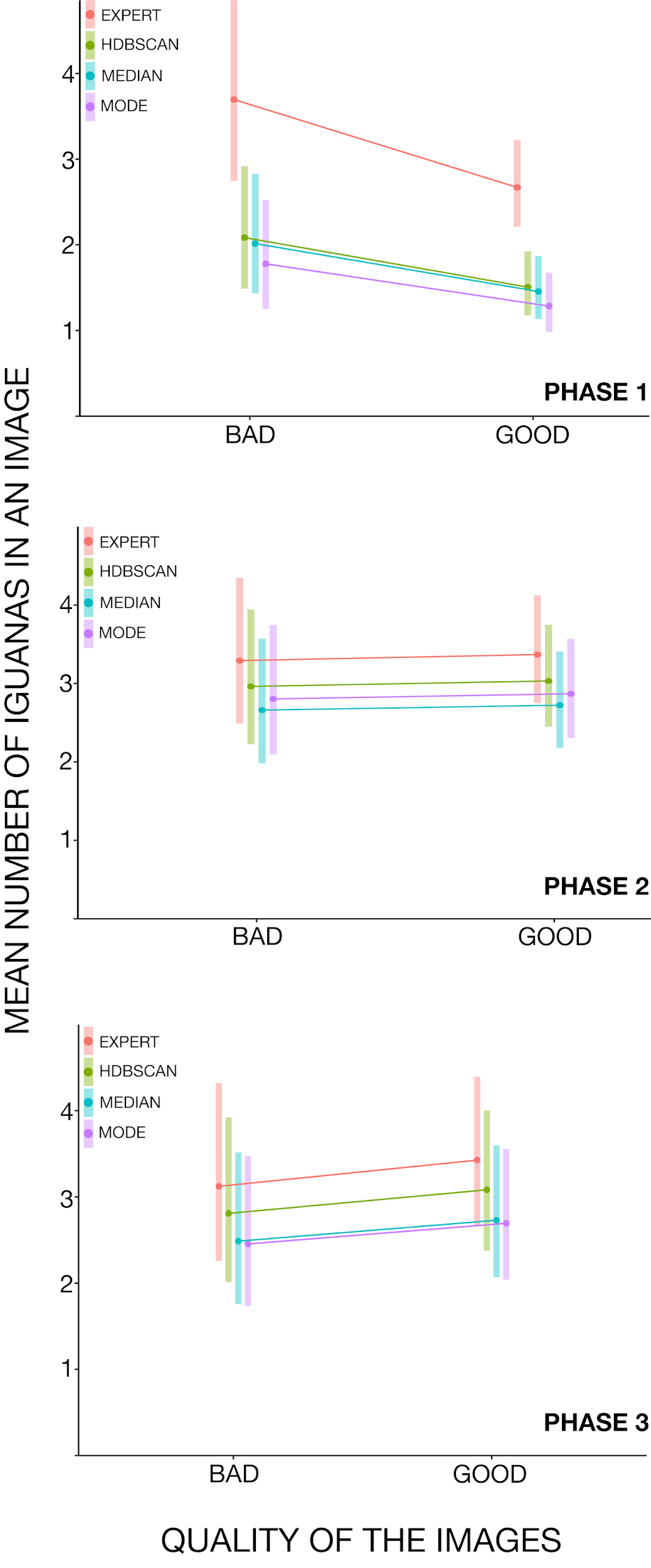


**Fig. S4.** Plots of the generalized linear models presented independently by phase when the factor ‘number of iguanas present in the image’ is added into the analysis, assessing differences amongst methods used to count marine iguanas from the GS dataset.


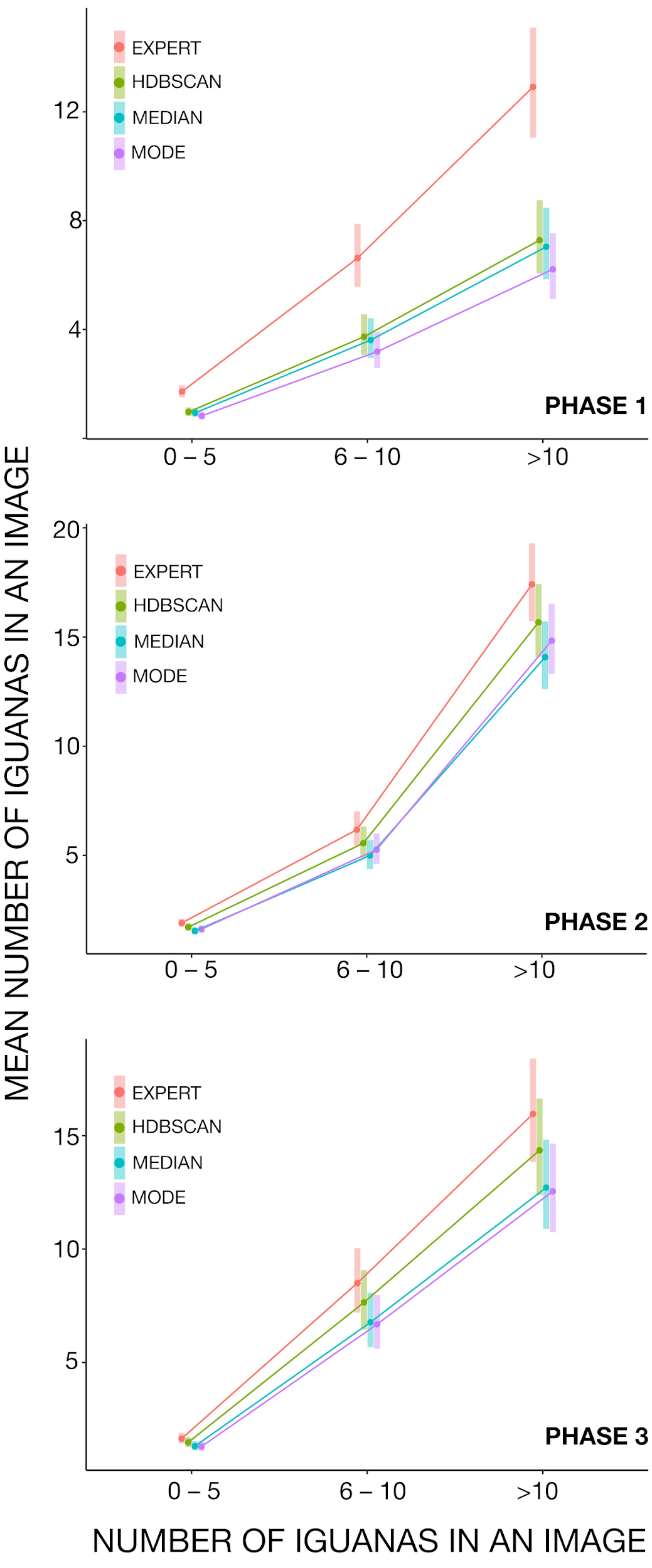


**Fig. S5.** Survey results of 110 volunteers regarding self-assessed difficulties in counting marine iguanas in the images in relation to the quality of the image and the number of iguanas present in the image.


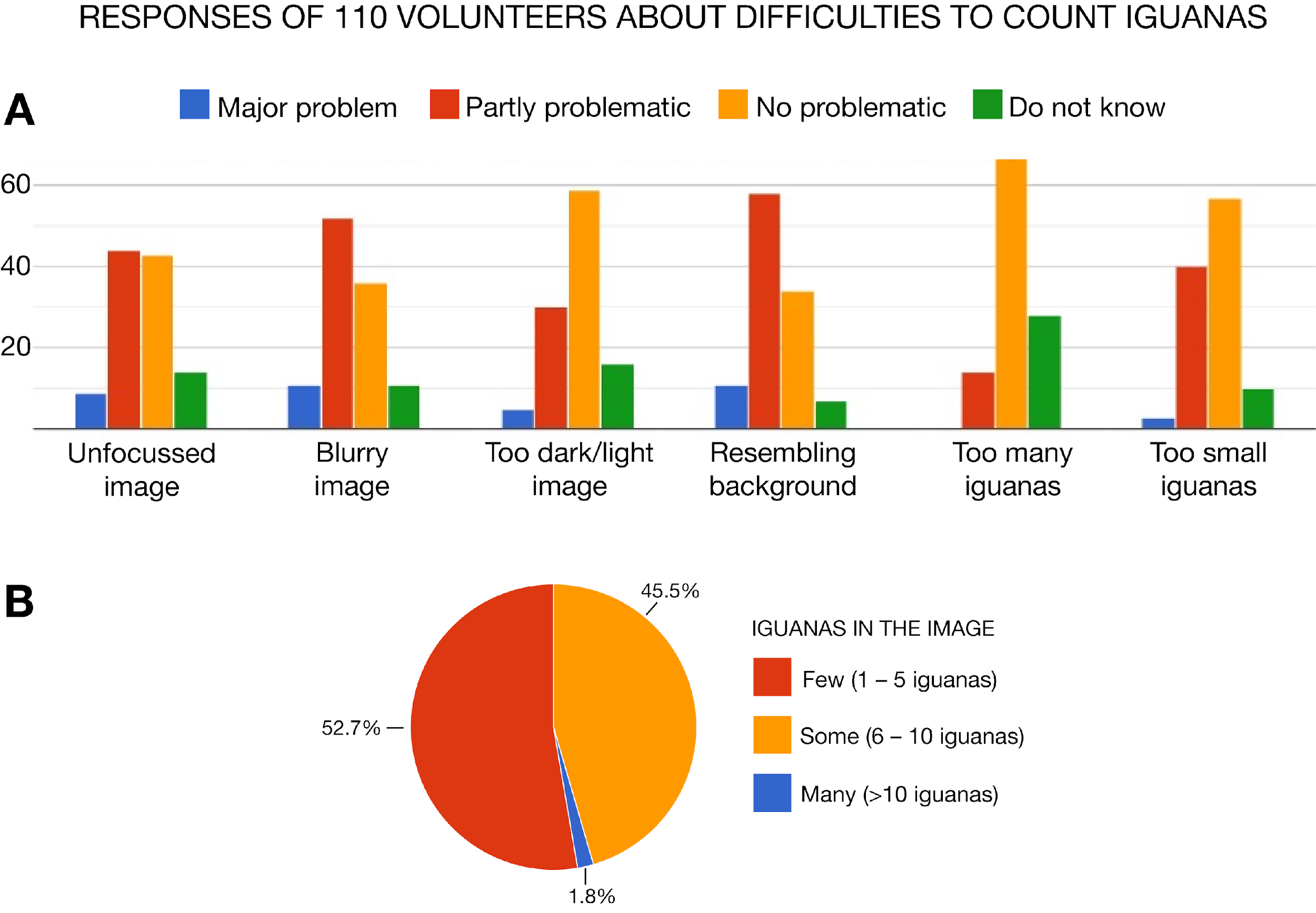


**Fig. S6.** Survey results of 110 volunteers regarding factors affecting their motivation to contribute further to the CS project.


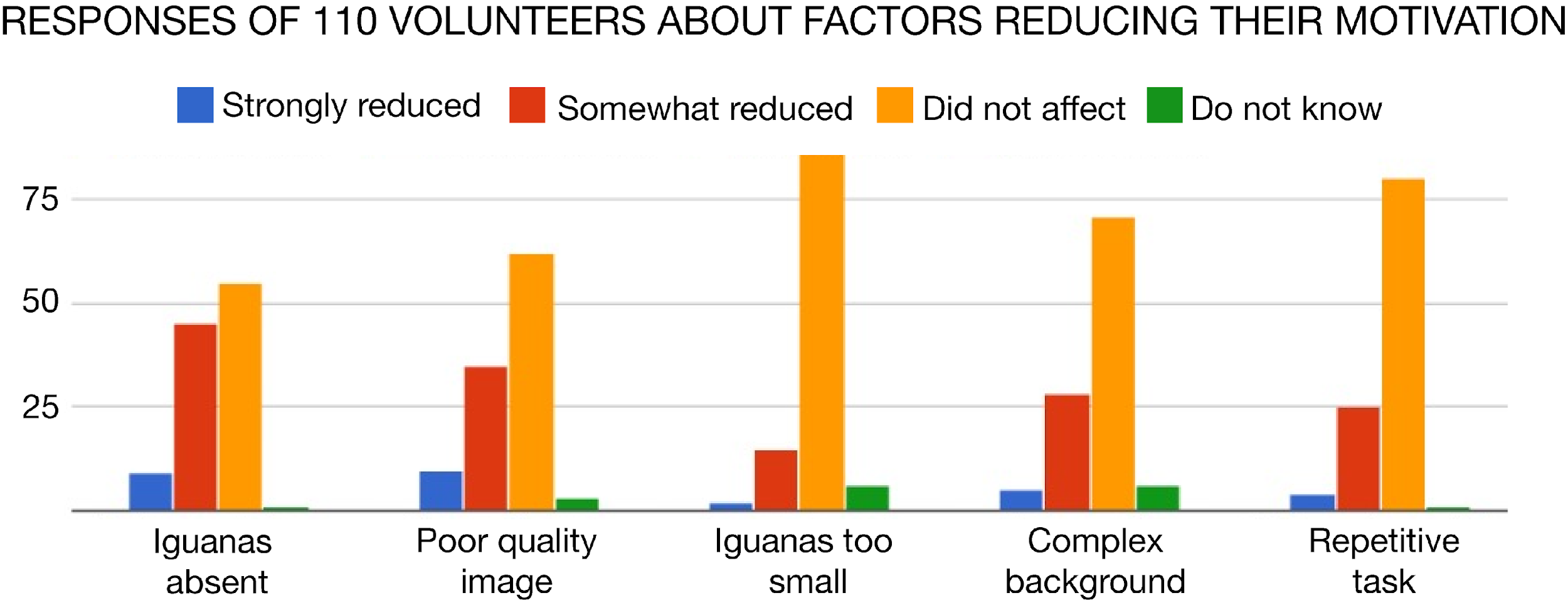


**Fig. S7.** Example image representing the number of iguanas counted by the expert compared to all the volunteers’ marks given to the same image. The HDBSCAN clustering method used the spatial marks to estimate the number of spatial clusters, which represent the aggregated number of iguanas counted by the volunteers. Here, a default minimum sample of 5 close marks forms a cluster.


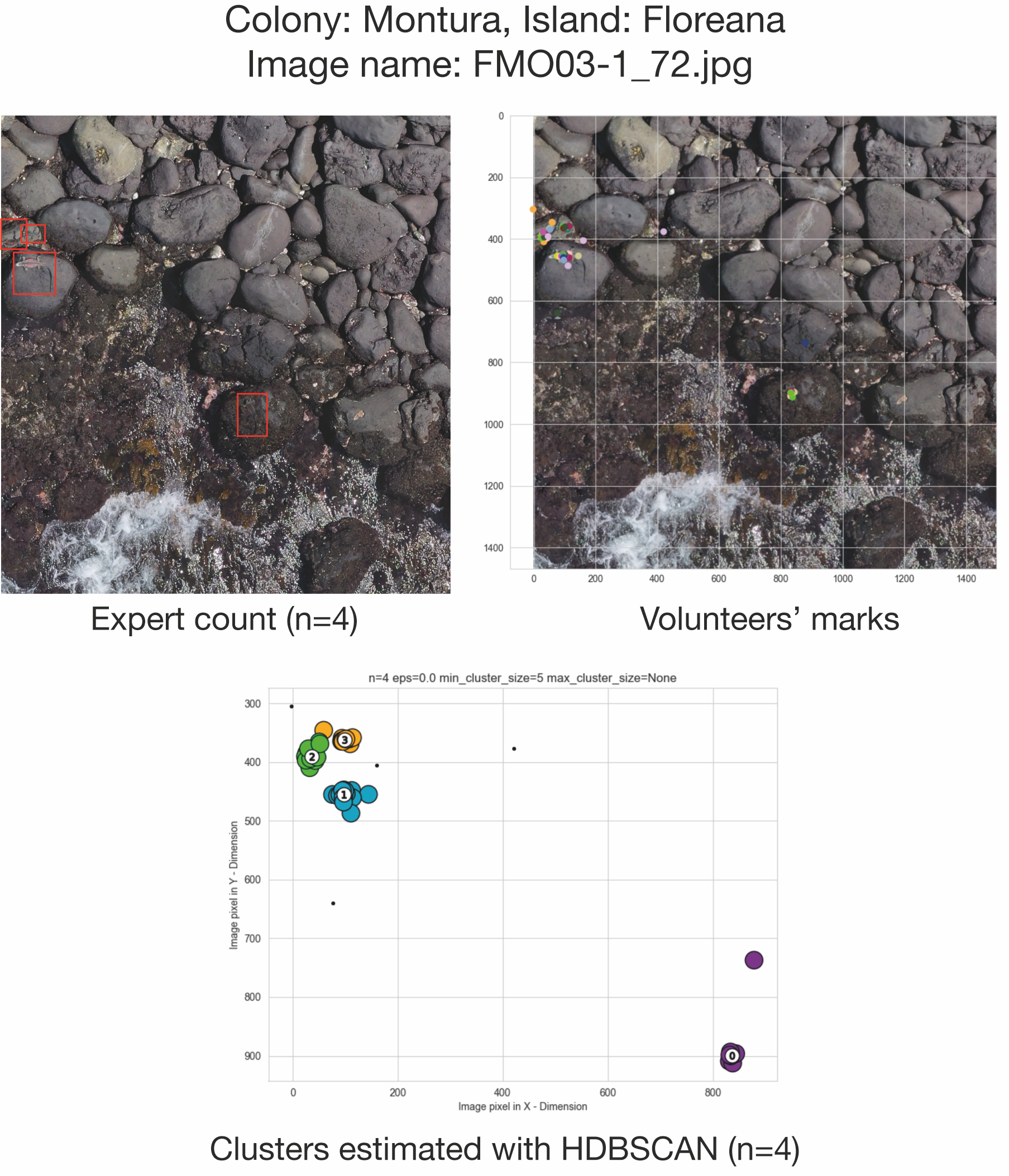


**Fig. S8.** Marine iguana counts of the Gold Standard dataset distribution. A) Histogram of the number of iguanas counted per image by the expert. B) Plot comparing expert counts vs CS counts aggregated with the HDBSCAN clustering method.


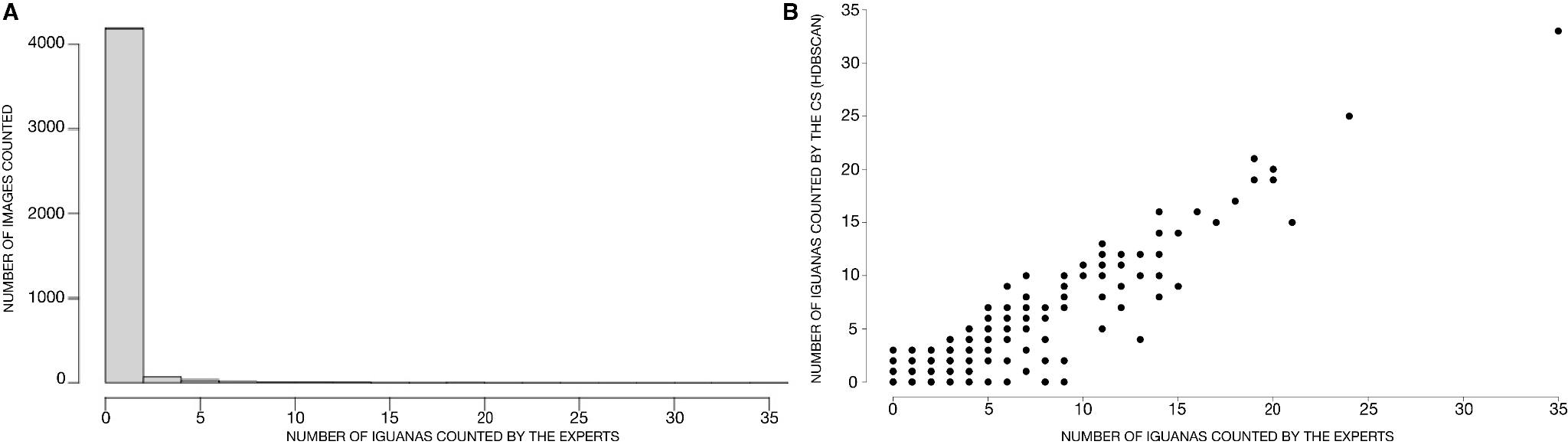


1. **Supplementary Tables**

**Table S1.** Number of images where CS counted equal to the experts, CS counted less than the experts, and CS counted more than the experts. Values in bold represent the best results.

| **Phase**  **(GS images)** | **Metric** | **CS = experts** | | **CS < experts**  **(underestimation)** | | **CS > experts**  **(overestimation)** | |
| --- | --- | --- | --- | --- | --- | --- | --- |
|  |  | **# images** | **%** | **# images** | **%** | **# images** | **%** |
| All (4345) | Median | 4096 | 94.3 | 205 | 4.7 | 44 | **1.0** |
|  | Mode | 4104 | 94.5 | 188 | 4.3 | 53 | 1.2 |
|  | HDBSCAN | 4114 | **94.7** | 175 | **4.0** | 56 | 1.3 |
|  | DBSCAN | 4042 | 93.0 | 198 | 4.6 | 105 | 2.4 |
| 1^st^ (2733) | Median | 2647 | **96.8** | 77 | 2.8 | 9 | **0.3** |
|  | Mode (max) | 2647 | **96.8** | 73 | **2.7** | 13 | 0.5 |
|  | HDBSCAN | 2644 | 96.7 | 76 | 2.8 | 13 | 0.5 |
|  | DBSCAN | 2624 | 96.0 | 81 | 2.9 | 28 | 1.0 |
| 2^nd^ (456) | Median | 361 | 79.1 | 72 | 16 | 23 | **5** |
|  | Mode | 368 | 80.7 | 63 | 13.8 | 25 | 5.5 |
|  | HDBSCAN | 376 | **82.5** | 56 | **12.2** | 24 | 5.3 |
|  | DBSCAN | 348 | 76.3 | 60 | 13 | 48 | 10.5 |
| 3^rd^ (1156) | Median | 1085 | 93.9 | 55 | 4.8 | 12 | **1.0** |
|  | Mode | 1086 | 93.9 | 51 | 4.4 | 15 | 1.3 |
|  | HDBSCAN | 1089 | **94.2** | 43 | **3.7** | 20 | 1.7 |
|  | DBSCAN | 1060 | 91.7 | 57 | 4.9 | 29 | 2.5 |

**Table S2**. Results of the logistical regression used to find an R-squared value that determines the best fit (closest) between volunteer counts and expert counts for all methods used to obtain volunteer counts. The bold value represents the best result.

| **Method** | **Model** | **Nagelkerdes R2** | **RMSE** | **Sigma** | **Score log** | **Performance score** |
| --- | --- | --- | --- | --- | --- | --- |
| HDBSCAN | glm | **0.912** | 3.295 | 1.200 | -1.963 | 80.00% |
| Median | glm | 0.891 | 3.614 | 1.295 | -2.040 | 50.57% |
| Mode | glm | 0.868 | 3.471 | 1.398 | -2.113 | 14.19% |

1. **Supplementary Methods**

**Text S1. Citizen science aggregating methods**

1. **Aggregating CS data with the median, mode, DBSCAN and HDBSCAN**

First, marks that depicted “partial iguanas” (i.e., individuals dissected when images were sliced in image preparation) were removed. We ran the median, mode, DBSCAN and HDBSCAN aggregating methods within the GS dataset where volunteers identified images to contain iguanas, using the minimum threshold approach identified for Task 1. Details on the methods are:

- 1. The **median** is the value in the middle of an ordered sample; we rounded it up when a decimal was obtained. The **mode** selects the most frequent value in a sample; if multiple values were equally frequent, we chose the highest one. These decisions were made after preliminary results indicated that volunteers tended to underestimate iguanas in the images.
  2. **DBSCAN** relies on at least two hyperparameters. The first is eps, which stands for Epsilon. This parameter refers to the threshold radius around any point. A point within the radius is considered a neighbour. The second is the minimum Points (min_points) parameter, which refers to the points required to form a dense region.

A grid search approach was chosen to find the best-fitting parameter set, using Silhouette scoring as the metric for optimal fit. The search space was eps = [0.01, 0.05, 0.1, 0.2, 0.3, 0.4, 0.5], min_samples = [3, 5, 8, 10].

Choosing eps and min_points effects found clusters. The optimal cluster count is found by maximizing the Silhouette score from each parameter. Two clusters are a minimum necessary to compare Silhouettes. This led to random results when sorting, when no two clusters could be found, resulting in worse outcomes.

- 1. **HDBSCAN** does not need an eps value set before the run. The default values, min_cluster_sizes = 5 and cluster_selection_epsilon = 0.0 were used.

Reference: Rousseeuw, P. J. 1987. Silhouettes: A graphical aid to the interpretation and validation cluster analysis. Journal of Computational and Applied Mathematics 20: 53–65.

<https://www.sciencedirect.com/science/article/pii/0377042787901257>

- 1. **An example from the colony is** El Miedo, in Santa Fe Island, phase 1. Image showing all volunteers’ marks and a table comparing the results of the different methods used (median, mode, DBSCAN and HDBSCAN) to aggregate data from volunteer classifications regarding the number of marine iguanas present in this image – these results illustrate why HDBSCAN (with a minimum threshold of 5 volunteers marking iguanas) generate better results, as the median selects the value in the middle of the sample while the mode chooses the most repeated value, excluding correct marks.
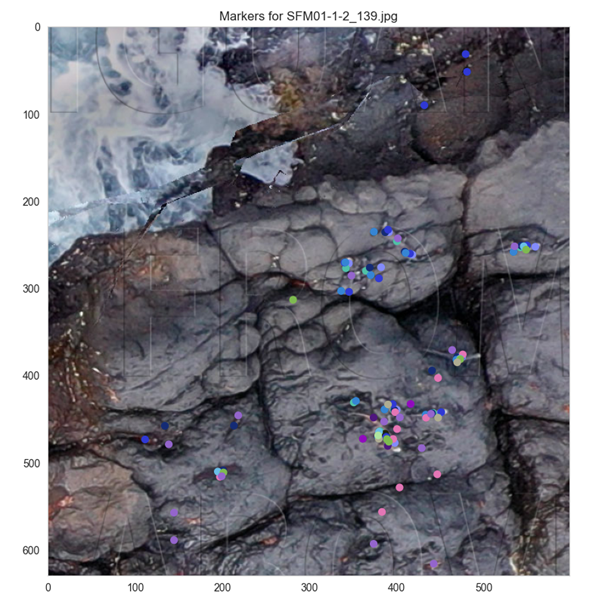


| **Image name** | **CS counts in ascendent order** | **Median** | **Mode** | **DBSCAN** | **HDBSCAN** | **Expert**  **count** |
| --- | --- | --- | --- | --- | --- | --- |
| SFM01-1-2_139.jpg | 1,1,1,2,2,4,4,5,9,9,10,11,12,14,15 | 5 | 1 | 5 | 8 | 11 |

- 1. **The code used to implement the aggregating methods into our data.**

The code is hosted on GitHub (<https://github.com/cwinkelmann/iguanas-from-above-zooniverse>), where its usage is explained. The two primary steps are:

- Extracting the data from the raw classifications file (available at: <https://github.com/cwinkelmann/iguanas-from-above-zooniverse/blob/main/Panoptes_Data_Prep.ipynb>) and prepare it for aggregation. The link to access the raw data is described in the Data Availability section of the paper.
- Aggregating the volunteers’ marks with the different methods tested (<https://github.com/cwinkelmann/iguanas-from-above-zooniverse/blob/main/Zooniverse_Clustering_all_panoptics.ipynb>)

**Text S2. Survey questions presented to our volunteers regarding their experiences with our project.**

**How many classifications** do you estimate you have made on this project?

- 1 – 100
- 101 – 500
- 501 – 1000
- > 1000

Please rate the following image features in terms of the **level of difficulty** they caused in finding the iguanas.

- Photo unfocused
- Image blurry/smeared
- Photo too dark/light
- Background too similar to iguanas
- Too many iguanas
- Iguanas too small

How much did the following aspects of the project **reduce your motivation** for classifying images?

- No iguanas in the images
- Poor quality image
- Iguanas too small
- Background too complex/similar to the iguanas
- Task repetitive

The iguanas were most **easily counted** in images with:

- Few iguanas (up to 5)
- Some iguanas (6–10)
- Many iguanas (> 11)
